# Can shear wave elastography predict the success of shock‑wave lithotripsy used in renal stones treatment? A prospective study

**DOI:** 10.1007/s00345-024-04855-z

**Published:** 2024-03-15

**Authors:** Mohamed Samir, Abdelrahim Galaleldine, Tarek El-Zayat, Noha Diaa Eldin, Mahmoud A. Mahmoud, Diaa Mostafa

**Affiliations:** Ain Shams University, Cairo, Egypt

**Keywords:** Shockwave lithotripsy, Renal stones, Hounsfield unit, Elastography, Stone density

## Abstract

**Objective:**

To evaluate the usage of shear wave elastography (SWE) in the prediction of the success rate of shock‑wave lithotripsy (SWL) treatment of renal stones.

**Patients and methods:**

In the present study, SWL was performed for 100 patients presented with renal stones in the duration from May 2022 to August 2023. The patients were divided into 2 groups SWL responders and non-responders. The study compared between the 2 groups in terms of baseline parameters of the patients as age, sex, body mass index (BMI), stone size, stone location, stone density (HU), skin-to-stone distance (SSD), the degree of hydronephrosis and the stone elastography values.

**Results:**

There was no statistically significant relation between stone-free rate and degree of obstruction (*p* = 0.628), stone size (*p* = 0.390) upper calyceal location (*p* = 0.17), middle calyceal location (*p* = 0.66), and renal pelvis location (*p* = 1.0). Nevertheless, a statistically significant relation was found as regards lower calyceal location, stone density (HU), and stone Elastography values using multivariate analysis.

**Conclusions:**

Measurement of stone density by shear wave elastography (SWE) can be used as an alternative to HU in decision-making before SWL. SWL success depends mainly on stone site, HU, and SWE values.

## Introduction

In 1980s, the use of shock wave lithotripsy (SWL) started to take place in the treatment of urolithiasis. It is considered as a viable management option for about 90% of adult stones [1]. According to the American Urological Association (AUA), European Association of Urology (EAU), Urological Association of Asia (UAA), Endourological Society (ES) and the National Institute of Health and Care Excellence (NICE) guidelines, SWL is advised for selected renal stone patients [2].

In the literature, the effectiveness of SWL widely varies from 46 to 91% [3]. So, it is vital to try to predict the outcome of SWL treatment. Thus, studies have investigated many parameters that can affect the success of SWL as stone factors (size, stone density (HU) and site), degree of hydronephrosis, efficacy of the machine, skin-to-stone distance (SSD) and body mass index (BMI) [4]. However, there is still no consensus regarding the use of these parameters to guide the selection of proper candidates for SWL [1] and it has been documented that renal stones patients are exposed to many CT scans with high doses of radiation [5].

Ultrasound Shear wave elastography (SWE) is a radiological modality used to measure tissue hardness [6]. SWE was investigated by many studies to assess its use in the measurement of tissue stiffness of body organs such as thyroid, breast, liver, prostate, and kidney [7, 8]. However, only a few studies have focused on its role in the evaluation of urinary stones [9, 10]. The aim of the present study was to evaluate the usage of SWE in the prediction of the success rate of SWL treatment as a radiation-free procedure with much lower cost in comparison to CT.

## Patients and methods

### Patients

The study was a prospective one conducted during the period from May 2022 to August 2023 in a single tertiary care hospital after approval of the ethical committee (ClinicalTrials.gov NCT05995652) All selected subjects gave written informed consent before participation in the study.

Patients aged > 18 years with a single radiopaque renal stone of 1–2 cm size for SWL were included in our study. While those with any urological abnormal anatomy, lower calyceal stone > 1.5 cm, DJ stents, impaired renal functions, severe hydronephrosis, concomitant ureteric stones, uncorrected coagulopathy, active urinary tract infection (UTI), pregnancy or single kidney were excluded.

#### Sample size

By PASS 11 for sample size calculation, setting power at 99%, alpha error at 5% and after reviewing previous study results [10] showed that among patients underwent SWL, the mean SWE in the group of successful SWL was lower than those in the group of failed SWL (7.9 + 2.2 and 17.9 + 10.2 respectively) [10]; Accordingly a sample size of at least 50 patients with kidney stones was required to achieve study objective.

### SWE technique

**Fig. 1:**
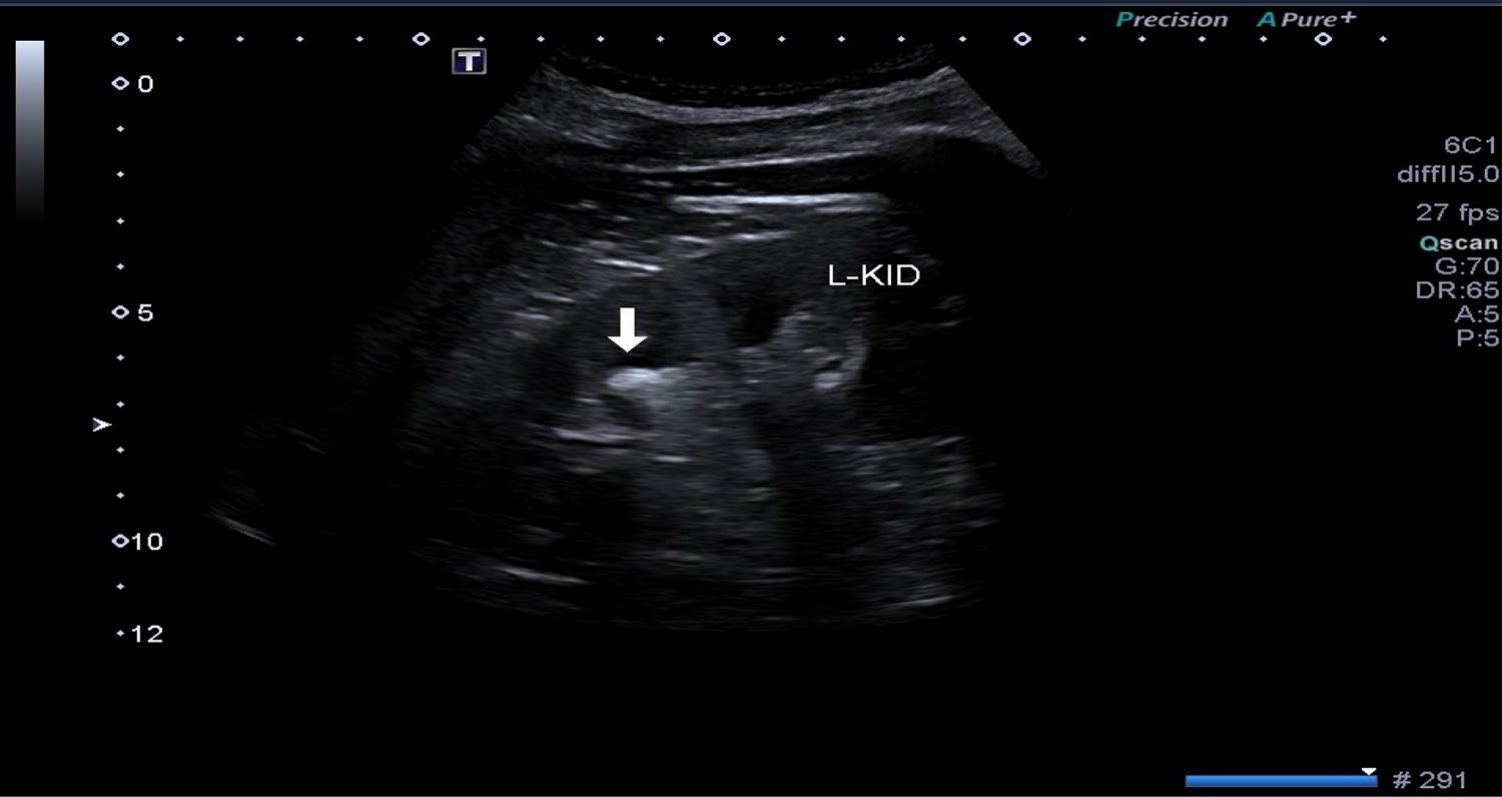
Measurement of left renal stone using SWE

One hundred patients who fulfilled the inclusion criteria were enrolled in our study. Non-contrast spiral computed tomography (NCCT) was performed in all cases using high-speed 64 MSCT helical (Toshiba, Tokyo, Japan). Grayscale renal ultrasonography (US) and SWE were done by the same consultant radiologist to calculate the elastography value of the stones using GE LOGIQ P9 (GE healthcare, Tokyo, Japan) device by a 9 MHz linear probe. Participants were examined while holding their breath and selecting the shortest distance to the chosen kidney in the right or left lateral decubitus or supine position. SWE measurements of the renal stone were expressed in kilopascals (kPa) (Fig. 1).

### SWL technique

SWL was carried out by the electromagnetic Dornier device (Dornier SII, Wessling, Germany) under proper analgesia. Patients were positioned supine and fluoroscopic guidance was used for stone localization. The session was terminated after 3500 shock waves were delivered or when stone fragments < 4 mm were seen. The power was increased gradually up to 4 kV and the shock wave rate was set at 80 /min. Patients were advised to drink liberal fluids and prescribed once daily alpha blocker (silodosin 8 mg tablet) and on-demand painkiller (diclofenac potassium).

### Follow‑up protocol

Patients were followed up at the outpatient clinic after 1 week by plain x-ray urinary tract (PUT) and ultrasonography (and if there is any suspicion NCCT) then after 1 month for the presence of residual stones. Residual stone fragments greater than 4 mm after 1 month of a single session of SWL were defined as failed treatment. So, the patients were divided into 2 groups SWL responders and non-responders.

The study compared between the 2 groups in terms of baseline parameters of the patients as age, sex, BMI, stone size, stone location, stone density (HU), SSD, the degree of hydronephrosis and the stone elastography values were compared between the 2 groups. The primary endpoint was to determine the role of SWE in the prediction of SWL outcome. The secondary endpoint was to investigate the correlation of HU and SWE.

### Statistical analysis

Continuous variables were either expressed as suitable means and standard deviations or medians and interquartile ranges. Categorical variables were presented as the counts and percentages in each category. Mann–Whitney *U* test was employed for continuous variables, and for categorical variables, chi-square and Fisher’s exact tests were used. By applying the receiver operating curve (ROC) analysis, we determined the optimal cut-off values of the stone density (HU) and the stone elastography values (kPa). Spearman correlations were used to assess the association between numerical variables. *p* < 0.05 was considered to be statistically significant. All these data analyses were conducted with the software SPSS 27.0 (SPSS Inc, Chicago, USA).

## Results

SWL was successful in 64% of subjects. There was no significant difference between responders and non-responders regarding demographic data except for BMI where non-responders showed higher mean BMI compared to responders, see Table 1.

**Table 1:** Demographic data of the studied patients

	Responders (64)		Non-responders (36)		*p* value
	Mean	± SD	Mean	± SD	
Age	41.25	12.10	44.00	12.00	0.277
BMI (kg/m^2^)	28.91	3.79	30.33	2.66	0.049
Sex					
Male	50	62.5%	30	37.5%	0.532
Female	14	70.0%	6	30.0%	

There was a significant difference between the responders and non-responders in relation to radiological data as stones’ density (HU), SWE and SSD with higher values in the non-responders. In addition, the lower calyceal position was significantly more in non-responders (*p* < 0.001). While there was no significant difference in stones’ size and backpressure grade, see Table 2.

**Table 2:** Comparison between responders and non-responders regarding radiological data

	Responders (64)		Non-responders (36)		*p* value
	Mean	± SD	Mean	± SD	
Size (mm)	12.73	2.35	13.19	2.91	0.390
Skin to stone distance (mm)	103.72	14.98	112.28	2.72	0.001
Stone density (HU)	807.77	164.75	1084.39	233.29	0.0001
SWE (kPa)	11.74	3.86	17.51	3.07	0.0001
Degree of obstruction					
No	34	58.6%	24	41.4%	0.339
Mild	28	70.0%	12	30.0%	
Moderate	2	100.0%	0	0.0%	
Upper calyx					
No	50	61.0%	32	39.0%	0.179
Yes	14	77.8%	4	22.2%	
Middle calyx					
No	53	63.1%	31	36.9%	0.666
Yes	11	68.8%	5	31.3%	
Pelvis					
No	32	64.0%	18	36.0%	1.0
Yes	32	64.0%	18	36.0%	
Lower calyx					
No	39	81.3%	9	48.1%	0.001
Yes	25	18.84%	27	51.9%	

After adjustment of all significant factors in univariate analysis, multivariate logistic regression analysis demonstrates that the following factors were predictive for stone-free rate:

- Lower calyx affection (*p* = 0.017, Adjusted Odds ratio = 5.8, 95% CI = 1.37–25.3).
- Stone density by HU (*p* = 0.017, Adjusted Odds ratio = 1.01, 95% CI = 1.002–1.01).
- Stone density by SWE (*p* = 0.001, Adjusted Odds ratio = 1.4, 95% CI = 1.2–1.85).

There was a highly statistically significant positive correlation between measurements of stone density by HU and SWE, see Table 3.

**Table 3:** Correlation between stone density by HU and by SWE

	Elastography by SWE
Stone density	
R*	0.459
*p*	0.0001
Sig	HS

*Pearson correlation

**Fig. 2:**
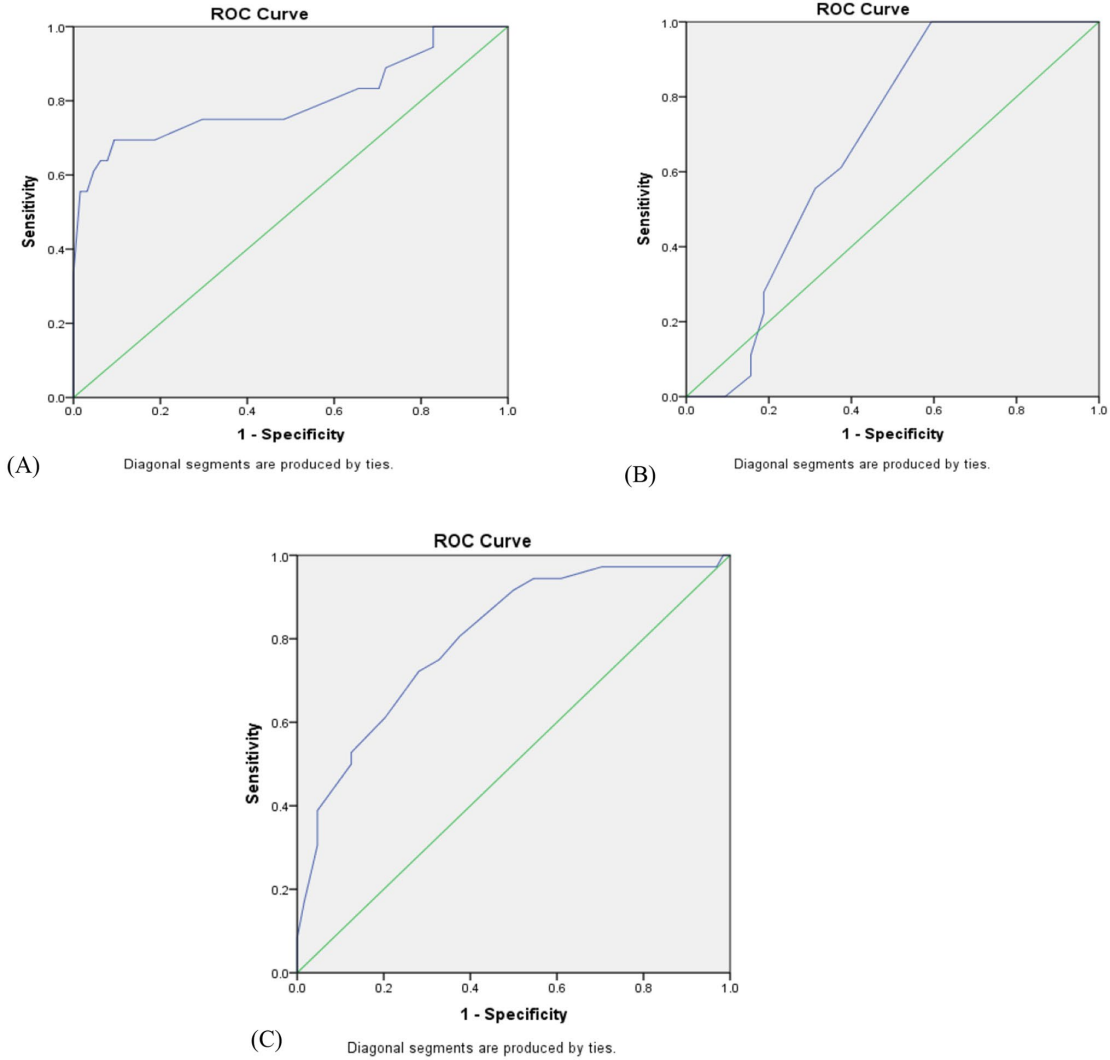
ROC curve: **a** stone density by HU (AUC = 0.8 & *p* value < 0.001), **b** SSD (AUC = 0.67 & *p* value = 0.038), c: SWE (AUC 0.80 & *p* value < 0.001)

ROC curve was used to determine the cutoff points with the highest sensitivity and specificity in the discrimination of treatment success was employed. SSD of value > 111.5 mm (55.6% sensitivity and 68.7% specificity), SWE of value ≥ 15.5 kPa (72.2% sensitivity, 71.8% specificity) and stone density of value of > 894HU (75% sensitivity, 70.3% specificity) were shown to be the best cutoff points, see Fig. 2.

## Discussion

The composition of urinary tract stones determines their hardness. Thus, calcium oxalate monohydrate and cystine stones have low SWL success rate as they are very hard [11]. However, stone components cannot be determined pre-SWL, so SWL cannot be avoided in such hard stones with unfavorable outcome. Only the HU value in CT can give an idea about the fragility of the stone. Low SWL success is linked to high HU [3, 12].

Unfortunately, CT imaging is associated with high cost and radiation exposure. Thus, studies are carried out to determine alternative parameters that can predict SWL outcome. Kraev et al. were the first to report the possibility of the use of SWE in determining the fragility of renal stones [9]. Then, Demir et al. documented the usability of SWE in the prediction of SWL success in their pilot study [10].

Concerning BMI, we concluded that it was a predictor of SWL success on univariate analysis but not independent predictor on multivariate analysis. The predictive value of BMI is debatable. Pareek et al., and El-Nahas et al., found it to be an independent variable of success [13, 14], while Ng et al., and Abdelhamid et al., did not [1, 15].

In terms of stone density (HU), we found a statistically significant difference between responders and non-responders with a cutoff value of > 894. Similarly, Perks et al. detected that stones < 900 HU were 6.2 times more likely to be treated successfully with SWL than were stones ≥ 900 HU [16], El-Assmy et al., reported that the HU cutoff value was > 1000 to determine SWL success [17]. Also, Hameed et al., stated that SWL outcome decreased in stones of HU > 1350 [18]. However, stone attenuation was not found to be an independent variable of SWL success by Wiesenthat et al. and Celik et al. on multivariate analysis [19, 20].

In our study, we found that lower calyceal location was an independent predictor of multivariate analysis of SWL success. It was seen in 25 (18.8%) subjects of the responders and 27 (51.9%) subjects of the non-responders (*p* = 0.001). In contrast, Abdelhamid et al., found lower calyceal location as a non-predictor variable [1].

In the present study, SSD was higher in the non-responders than responders (*p* < 0.001) with a cutoff value of > 111.5 mm. In agreement with this, Elawady et al., reported the cutoff value of SSD that predicted SWL success was 86 mm [21]. Also, Waqas et al. found that 100 mm was the suitable threshold value for SSD beyond which stone disintegration decreased [22]. Park et al. concluded that SSD was the only important factor for the prediction of SWL outcome and they explained that by the expected loss of shock waves energy on passing through the increased body fat percent with higher SSD [23]. While Geng et al., and Ng et al., did not find SSD as an independent variable of SWL success in their studies [15, 24].

Regarding SWE, we found that the mean SWE value was 11.74 ± 3.86 kPa in the responders, while it was 17.51 ± 3.07 kPa in the non-responders. This difference was highly statistically significant (*p* < 0.001), and the best cutoff value of SWE was ≥ 15.5 kPa to determine SWL success. Similarly, Demir et al., stated that the difference in SWE values between patients with successful SWL and patients with failed SWL was statistically significant (*p* < 0.05), They attributed that to the softness of stones with lower SWE [10].

In this study, it is noted that there is a correlation between the measurements of stone density by HU and SWE. This was also demonstrated by Demir et al. [10]. We believe SWE can replace HU in the prediction of SWL outcome. It will be helpful especially in avoiding radiation exposure in pediatric population.

Our study is small-scale as it has been conducted on relatively small number of participants and no chemical stone analysis. We are in need of further studies to validate our results. However, this study defined the role of SWE in the prediction SWL success for renal stones. Besides, it established the correlation between the measurement of stone density by SWE and HU.

## Conclusions

SWE is an additive helpful cheap tool with less radiation exposure than CT for prediction of SWL outcome. Lower HU, SWE and site of renal stones are reliable predictors of SWL success. Furthermore, there is a positive correlation between measurements of stone hardness by HU and stone density by SWE.

## Data Availability

Data is available on request.

## References

[1] Abdelhamid M, Mosharafa AA, Ibrahim H, Selim HM, Hamed M, Elghoneimy MN, Salem HK, Abdelazim MS, Badawy H (2016) A prospective evaluation of high-resolution CT parameters in predicting extracorporeal shockwave lithotripsy success for upper urinary tract calculi. J Endourol 30(11):1227–1232 27597174 10.1089/end.2016.0364

[2] Colakerol A, Suzan S, Temiz MZ, Gonultas S, Aykan S, Ozsoy S, Kucuk SH, Yuruk E, Kandırali E, Semercioz A (2022) Tissue neutrophil elastase contributes to extracorporeal shock wave lithotripsy-induced kidney damage and the neutrophil elastase inhibitor, sivelestat, attenuates kidney damage with gratifying immunohistopathological and biochemical findings: an experimental study. Urolithiasis 50(1):103–112 34778918 10.1007/s00240-021-01287-x

[3] Lee HY, Yang YH, Lee YL, Shen JT, Jang MY, Shih PM, Wu WJ, Chou YH, Juan YS (2015) Noncontrast computed tomography factors that predict the renal stone outcome after shock wave lithotripsy. Clin Imaging 39(5):845–850 25975631 10.1016/j.clinimag.2015.04.010

[4] Erkoc M, Bozkurt M, Besiroglu H, Canat L, Atalay HA (2021) Success of extracorporeal shock wave lithotripsy based on CT texture analysis. Int J Clin Pract 75(11):e14823 34491588 10.1111/ijcp.14823

[5] Katz SI, Saluja S, James A (2006) Brink radiation dose associated with unenhanced CT for suspected renal colic: impact of repetitive studies. AJR 186:1120–1124 16554590 10.2214/AJR.04.1838

[6] Turkay R, Inci E, Bas D, Atar A (2018) Shear wave elastographic alterations in the kidney after extracorporeal shock wave lithotripsy. J Ultrasound Med 37(3):629–634 29027695 10.1002/jum.14415

[7] Bamber J, Cosgrove D, Dietrich CF et al (2013) MEFSUMB guidelines and recommendations on the clinical use of ultrasound elastography, part1: basic principles and technology. Ultraschall Med 34:169–184 23558397 10.1055/s-0033-1335205

[8] Samir AE, Allegretti AS, Zhu Q et al (2015) Shear wave elastography in chronic kidney disease: a pilot experience in native kidneys. BMC Nephrol 16:119 26227484 10.1186/s12882-015-0120-7 PMC4521488

[9] Kraev G, Rudenko VI, Amosov AV, Krupinov GE, Ganzha TM (2016) Clinical implications of shear wave ultrasound elastography for evaluation of urinary stones. Urologiia (5):16–20 28248014

[10] Demir M, Dere O, Yağmur İ, Katı B, Pelit ES, Albayrak İH, Çiftçi H (2021) Usability of shear wave elastography to predict the success of extracorporeal shock-wave lithotripsy: prospective pilot study. Urolithiasis 49(3):255–260 33104861 10.1007/s00240-020-01221-7

[11] Dretler SP (1988) Stone fragility: a new therapeutic distinction. J Urol 139(5):1124–1127 3361657 10.1016/s0022-5347(17)42801-1

[12] Xun Y, Li J, Geng Y et al (2018) Single extracorporeal shockwave lithotripsy for proximal ureter stones: can CT texture analysis technique help predict the therapeutic effect? Eur J Radiol 107:84–89 30292278 10.1016/j.ejrad.2018.08.018

[13] Pareek G, Hedican S, Lee F, Nakada S (2005) Shock wave lithotripsy success determined by skin-to-stone distance on computed tomography. Urology 66(5):941–944 16286099 10.1016/j.urology.2005.05.011

[14] El-Nahas AR, El-Assmy AM, Mansour O, Sheir KZ (2007) A prospective multivariate analysis of factors predicting stone disintegration by extracorporeal shock wave lithotripsy: the value of high-resolution noncontrast computed tomography. Eur Urol 51(6):1688–1693 17161522 10.1016/j.eururo.2006.11.048

[15] Ng CF, Siu DY, Wong A, Goggins W, Chan ES, Wong KT (2009) Development of a scoring system from noncontrast computerized tomography measurements to improve the selection of upper ureteral stone for extracorporeal shock wave lithotripsy. J Urol 181(3):1151–1157 19152949 10.1016/j.juro.2008.10.161

[16] Perks AE, Schuler TD, Lee J, Ghiculete D, Chung D-G, Honey RJDA et al (2008) Stone attenuation and skin-to-stone distance on computed tomography predicts for stone fragmentation by shock wave lithotripsy. Urology 72(4):765–769 18674803 10.1016/j.urology.2008.05.046

[17] El-Assmy A, Abou-el-Ghar ME, El-Nahas AR, Refaie HF, Sheir KZ (2011) Multidetector computed tomography: role in determination of urinary stones composition and disintegration with extracorporeal shock wave lithotripsy an in vitro study. Urology 77:286–290 20719366 10.1016/j.urology.2010.05.021

[18] Hameed DA, Elgammal MA, ElGanainy EO, Hageb A, Mohammed K, El-Taher AM, Mostafa MM, Ahmed AI (2013) Comparing non contrast computerized tomography criteria versus dual X-ray absorptiometry as predictors of radioopaque upper urinary tract stone fragmentation after electromagnetic shockwave lithotripsy. Urolithiasis 41:511–515 23907170 10.1007/s00240-013-0596-1

[19] Wiesenthal JD, Ghiculete D, D’A Honey RJ, Pace KT (2010) Evaluating the importance of mean stone density and skin-to-stone distance in predicting successful shock wave lithotripsy of renal and ureteric calculi. Urol Res 38:307–313 20625891 10.1007/s00240-010-0295-0

[20] Celik S, Bozkurt O, Kaya FG, Egriboyun S, Demir O, Secil M, Celebi I (2015) Evaluation of computed tomography findings for success prediction after extracorporeal shock wave lithotripsy for urinary tract stone disease. Int Urol Nephrol 47:69–73 25311505 10.1007/s11255-014-0857-0

[21] Elawady H, Mahmoud MA, Samir M (2022) Can we successfully predict the outcome for extracorporeal shock wave lithotripsy (ESWL) for medium size renal stones? A single-center experience. Urologia 89(2):235–239 33985373 10.1177/03915603211016355

[22] Waqas M, Saqib IU, Imran Jamil M et al (2018) Evaluating the importance of different computed tomography scan-based factors in predicting the outcome of extracorporeal shock wave lithotripsy for renal stones. Investig Clin Urol 59(1):25–31 29333511 10.4111/icu.2018.59.1.25 PMC5754579

[23] Park BH, Choi H, Kim JB et al (2012) Analyzing the effect of distance from skin to stone by computed tomography scan on the extracorporeal shock wave lithotripsy stone-free rate of renal stones. Korean J Urol 53:40–43 22323973 10.4111/kju.2012.53.1.40 PMC3272555

[24] Geng JH, Tu HP, Shih PM, Shen JT, Jang MY, Wu WJ, Li CC, Chou YH, Juan YS (2015) Noncontrast computed tomography can predict the outcome of shockwave lithotripsy via accurate stone measurement and abdominal fat distribution determination. Kaohsiung J Med Sci 31(1):34–44 25600918 10.1016/j.kjms.2014.10.001 PMC11916009

